# Translation, cultural adaptation and validation of Patient Satisfaction with Pharmacist Services Questionnaire (PSPSQ) 2.0 into the Arabic language among people with diabetes

**DOI:** 10.1371/journal.pone.0298848

**Published:** 2024-06-27

**Authors:** Basmah Albabtain, Vibhu Paudyal, Ejaz Cheema, Ghada Bawazeer, Abdulaziz Alqahtani, Ahmed Bahatheq, Farag Shuweihdi, Muhammad Abdul Hadi

**Affiliations:** 1 Department of Pharmacy Practice, College of Pharmacy, Princess Nourah Bint Abdulrahaman University, Riyadh, Saudi Arabia; 2 School of Pharmacy, Institute of Clinical Sciences, University of Birmingham, Birmingham, United Kingdom; 3 School of Pharmacy, University of Management and Technology, Lahore, Pakistan; 4 Department of Clinical Pharmacy, College of Pharmacy, King Saud University, Riyadh, Saudi Arabia; 5 Pharmacy Services, King Saud University Medical City, Riyadh, Saudi Arabia; 6 Saudi Innova Healthcare Company, Riyadh, Saudi Arabia; 7 Leeds Institute of Health Sciences, University of Leeds, Leeds, United Kingdom; 8 Department of Clinical Pharmacy and Practice, College of Pharmacy, QU Health, Qatar University, Doha, Qatar; Qatar University College of Nursing, QATAR

## Abstract

**Background:**

Understanding patient satisfaction is key to advancing pharmacy services and improving health outcomes. There is a lack of a translated and psychometrically validated tool in the Arabic language to measure patient satisfaction with pharmacy services.

**Objective:**

To translate the English version of the PSPSQ 2.0 into Arabic language, culturally adapt, and verify its reliability and validity.

**Setting:**

A community pharmacy in Riyadh, Saudi Arabia.

**Method:**

A cross-sectional study was conducted between April 2021 and June 2022 among patients with diabetes attending a community pharmacy. The International Society for Pharmacoeconomics and Outcomes Research good practice guidelines for linguistic translation and cultural adaptation were used to translate and culturally adapt the English version of PSPSQ 2.0 into Arabic. The Arabic version of PSPSQ 2.0 was subjected to factor analysis using principal component analysis with varimax rotation to evaluate its validity and Cronbach’s alpha was used to assess the reliability of PSPSQ 2.0.

**Results:**

A total of 129 (68.2% male, and mean age 50 (SD: 11.9) years) patients with diabetes participated in the study. The analysis was undertaken for the items in each of the three domains of PSPSQ 2.0: quality of care, interprofessional relationship and overall care. Exploratory factor analysis revealed validity of 92.7%, 80.5% and 96.2%, respectively. The Arabic version of PSPSQ 2.0 had high internal consistency with Cronbach’s alpha scores 0.99, 0.95 and 0.98 for the three measured domains, respectively. The sample adequacy was 0.924.

**Conclusion:**

The PSPSQ 2.0 was successfully translated and culturally adapted into the Arabic language and had acceptable validity and reliability to measure patient satisfaction with services provided by pharmacists in community pharmacies.

## Introduction

Patient satisfaction with healthcare services has gained increased attention as a valuable indicator to measure and improve the quality of healthcare services [1, 2]. It is used as one of the benchmark indicators to evaluate and identify specific areas of the service offered by pharmacists in practice settings that need improvement [3]. Patient satisfaction evaluation may help identify patients’ needs, perceptions, concerns, and any shortcomings associated with the healthcare system [4]. Furthermore, there is an emphasis on measuring patient satisfaction as an outcome of care [5]. Patient satisfaction is often referred to as a humanistic or patient-reported outcome that, in addition to clinical and economic outcomes, serves as an important determinant of the success, viability and sustainability of healthcare services [6].

Previous studies have reported an association between positive health outcomes and patient satisfaction. Patients with high service satisfaction are likely to comply with treatment, take an active role in their own care, continue using medical care services, treasure their relationship with their healthcare providers, and increase their adherence to medication [4, 7–11]. Although patient satisfaction is such a widely investigated construct, there is still a lack of a universally accepted definition [12] and satisfaction has been defined in various ways [7, 13, 14]. However, by reviewing the existing definitions, common criteria, three general components can be identified: first, consumer satisfaction is an emotional or cognitive response; second, the response pertains to a particular focus such as expectations, product, and consumption experience; third, the response occurs at a particular time e.g. after consumption and after choice [15]. The absence of a universally accepted definition of satisfaction complicates cross-study comparisons, challenges the development of standardized measurement tools, and hinders routine use as a quality indicator of healthcare services [12].

Several research instruments are available for assessing patient satisfaction with pharmacy services in various healthcare settings such as the Pharmaceutical Care Satisfaction Questionnaire, Patient Satisfaction Questionnaire, Pharmacy Encounter Survey, Pharmaceutical Care Questionnaire, and Patient Satisfaction Scale [16]. A psychometric evaluation was conducted on these instruments so that they might be used as quality assurance tools, and they have proven to be valid and reliable in evaluating pharmaceutical care. However, these instruments often suffer from several limitations including: poor face and content validity, inappropriate psychometric testing, limited reproducibility, measuring constructs other than patient satisfaction and limited to use in a specific setting. Subsequently, researchers are often reluctant to use these scales [1].

Historically, in 1983, Ware and colleagues developed the Patient Satisfaction Questionnaire (PSQ), 55 Likert-type items related to 9 domains, to measure patient satisfaction in health care services including pharmacy-based services [17]. In 2015, Sakharkar et al. developed a validated and reliable instrument "Patient Satisfaction with Pharmacist Services" (PSPSQ 2.0) as a tool to evaluate the quality and patient satisfaction with pharmacists-delivered clinical services [1], based on Ware’s framework and building upon the work of MacKeigan and Larson, and others [1, 16, 17].

The PSPSQ 2.0 has several advantages including relative ease of use in practice, simple scoring method, validity and reliability in different settings [1, 2, 18]. Additionally, satisfaction scores can be compared over time with those of other pharmacies or within a single pharmacy for the purpose of improving services. The PSPS 2.0 instrument has been translated and cross-culturally adapted into other languages, and its measurement properties have been reported to be satisfactory [2, 18]. PSPSQ 2.0 has not yet been translated into Arabic and psychometrically validated for patients with chronic conditions who received pharmacy services.

Arabic is one of the six official languages of the United Nations, as it is the fifth most spoken language in the world. Arabic is primarily spoken in different geographical regions of different countries (Arabian Peninsula, North Africa, and the Middle East) that are similar in terms of culture, healthcare beliefs, cultural influence, etc. Thus, all of these nations stand to gain significantly from the translated, culturally adapted, and validated questionnaire [19]. Given the rapid growth currently being witnessed in the Arab world in the scope of pharmacy services, the availability of an Arabic version of PSPSQ 2.0 would assist in improving pharmacy services by measuring patient satisfaction among Arabic-speaking people. To facilitate and suit the Arabic population context, translation, cultural adaptation, and validation into Arabic are required. As a result, the purpose of this research was to translate the PSPSQ 2.0 into Arabic, adapt it to the culture, and test its reliability and validity in a community setting.

## Methods

### Study design and settings

This cross-sectional validation study is part of a larger mixed-methods research project which focused on the development, implementation and evaluation of a community pharmacy based medication therapy management (MTM) program in Saudi Arabia [20]. The study used the PSPS 2.0 tool developed by Sakharkar et al. to translate it to Arabic, culturally adapt, and test its reliability and validity in a community setting [1] **(S1 File).**

The research was conducted at one of the Health Kingdom Community Pharmacy chains in Saudi Arabia, a medium-sized private pharmacy group. The community pharmacy is located east of Riyadh and is affiliated with a private polyclinic medical center and provide routine community pharmacy services to patients and carers. A private room was designated within the pharmacy for the MTM service. The MTM service was the first to be established in a community pharmacy in Saudi Arabia [20].

### Study population, inclusion and exclusion criteria

For the pilot study, eligible patients visiting the community pharmacy to refill their regular medicines were conveniently approached and enrolled. During the actual (non-pilot) study period, eligible patients who visited the health care center were informed about the study by their physicians and were selected randomly.

To adequately represent the target population, the eligibility criteria for both pilot and full-phase studies were similar. Patients who met the following inclusion criteria were included in the study: (a) has uncontrolled diabetes; (b) is at least 18 years old; (c) is able to understand Arabic; and (d) has an active status with the medical center. Participants who were diagnosed with severe mental illness or dementia or significant cognitive impairment, or gestational diabetes, and patients with unstable acute complications or illnesses, were excluded from the study. Detailed study methodology has been previously published [20].

For the psychometric evaluation of questionnaires, an item-response theory (IRT) was used to calculate the sample size [21]. Numerous studies have proposed different sample sizes based on different item-to-respondent ratio, 1:(5–10) up to a total of 300 [22, 23]. For this study, the 1:5 item-to-respondent ratio or a minimum of 100 samples is considered an appropriate sample size and required in conducting a factor analysis [24–26].

### Research instruments

#### Original PSPSQ 2.0

PSPSQ 2.0 developed by Shahakar et al., has been evaluated psychometrically [1]. It is a validated and reliable instrument for measuring patient satisfaction with pharmacist services **(S1 File).** This self-reported tool consists of 20 items divided across three domains: quality of care (10 items), patient-pharmacist relationship (6 items), and overall satisfaction (4 items). It assesses patients’ level of agreement with a 4-point, Likert-type scale (strongly agree, agree, disagree, and strongly disagree). Overall, it takes an average of ten minutes to complete. The mean level of satisfaction of patients is calculated by averaging their ratings for 20 parameters of measuring satisfaction. The resulting mean is interpreted by considering the closest Likert scale to it [1].

#### Demographic questionnaire

The questionnaire was developed for the main study [20], comprising of items that explored demographic and related information of patients: age, gender, nationality, educational qualification, insurance coverage, family history, and financial income. Medication-related questions included types of chronic illness, HbA1c level, length of diagnosis, and the number of medications.

#### Translation, cultural adaptation, and validation

**Fig 1:**
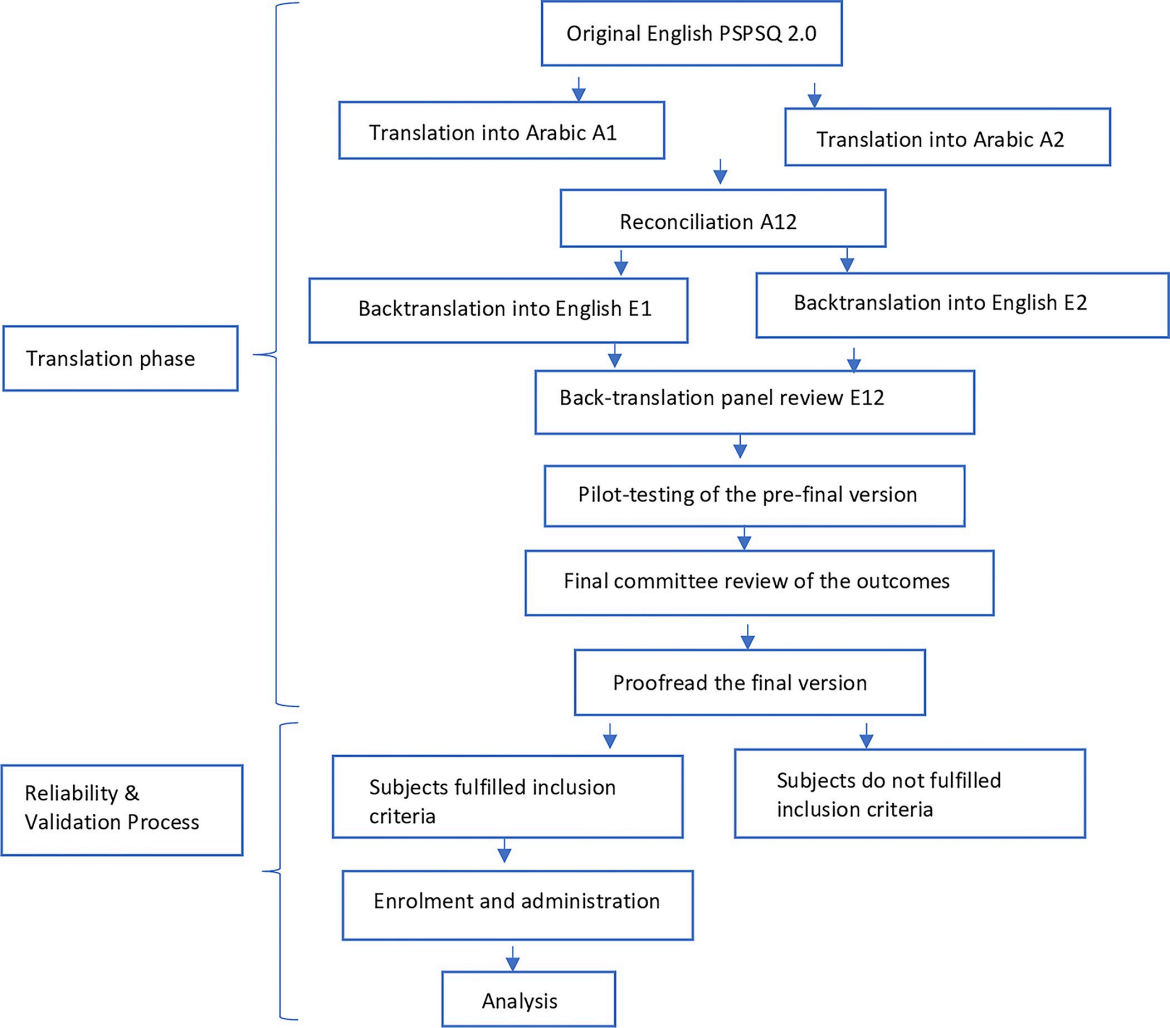
Overview of the process of translation, cross-cultural adaptation and validation of the PSPSQ 2.0.

The methods for translation, cultural adaptation, validation, and reliability are described briefly under respective subheadings below. The process of translation, cultural adaptation, and validation of this project is summarized in Fig 1.

#### Step 1—Translation procedure and cultural adaptation

The process of translation and cultural adaptation of PSPSQ 2.0 followed the standard protocol of the International Society for Pharmacoeconomics and Outcomes Research (ISPOR) Good Practice Guidelines for linguistic and cultural adaptation and validation [27]. The translation and cross-cultural adaptation process was done in seven different stages **(Fig 1 and Table 1).**

**Table 1:** The translation and cross-cultural adaptation process stages.

Stages	Description
**Stage 1: forward translation**	Two bicultural, native Arabic speakers (GA and AB) with a good command of English translated independently the English version of PSPSQ 2.0 into the Arabic language. One of the translators was academic pharmacist (GA) and another was a medical physician (AB).
**Stage 2: reconciliation**	The two Arabic forward translated versions were evaluated, revised and critically appraised by the main author along with the two forward translators in order to merge two forward translations into one reconciled version of the translated questionnaire (A12).
**Stage 3: backward translation**	The reconciled version (A12) was then back-translated into the source language (English) by another pair, independent back-translators (MY and GK) one of the translators was clinical pharmacist (MY) and another was an academic pharmacist (GK). The translators were bicultural, native Arabic speakers who were also fluent in English. The back translators were blinded to the original English version, and they were asked to report any sort of difficulty during translation.
**Stage 4: back-translation panel review of the backtranslation**	Both back-translations (E1 and E2) were compared and reviewed by the study team for cultural adaptation against the original English version to ensure the conceptual equivalence of the translation and identify any discrepancies. Necessary modifications were made to the items to generate reconciled version (E12). Then, the reconciled version (E12) was sent via email to the questionnaire developer for review and feedback. After reaching agreement, approved preliminary of the translated Arabic version “intermediate version” of PSPSQ 2.0 was used in the pilot study.
**Stage 5: cognitive debriefing**	Upon ensuring consistency in the translated version and the original English version, the preliminary Arabic version of PSPSQ 2.0 was pre-tested in a small group of 10 conveniently selected patients. To adequately represent the target population for the main study, the subjects were selected from the same community pharmacy that have MTM clinic. Patients were asked to evaluate the questionnaire for intelligibility, appearance, clarity, and wording, also encouraged to give suggestions for improvements. Aiming to assess the level of comprehensibility and cognitive equivalence of the translation and test any translation alternatives, participants were asked to highlight any items that may be inappropriate at a conceptual level and identify any other issues that cause confusion on understanding the questions.
**Stage 6: review of cognitive debriefing results and finalization**	The authors reviewed and incorporated the findings that results from cognitive debriefing to improve the performance of the translation and identifies translation modifications necessary for improvement. Following agreement on changes, the translation of Arabic version of PSPSQ 2.0 was finalized.
**Stage 7: Proofreading**	The authors checked the final translation and corrected any errors in spelling, diacritical, grammatical, or other errors which have been missed during the translation process. Then, the final revised pre-tested version of the translated questionnaire was subjected to psychometric analysis **(S2 File).**

#### Step 2—Validation and reliability analysis

*Sampling adequacy and sphericity*. Before the performance of exploratory for its appropriateness in the factor analysis, the sampling adequacy was analysed using the Kaiser-Meyer-Olkin (KMO). KMO has to be more than 0.5 to be considered acceptable. On the other hand, Bartlett’s test of sphericity was completed. It should be significant (p <0.05) which indicates that it is appropriate and worth continuing with factor analysis as there are relationships to investigate [28–30].

*Face validity*. The face validity of the PSPSQ 2.0 was performed by collecting feedback from the participants of the pilot study (n = 10) and subject experts. During the pilot study, the self-administered intermediate version was administered to ten patients comprised of native Arabic speakers visiting the community pharmacy (Health Kingdom Community Pharmacy). Participants were asked to respond to the questionnaires and provide comments on the clarity, appearance, and suitability of the tool to measure patients’ satisfaction. They were approached for any suggestions to improve the quality of the questionnaire. None of the participants reported any disagreement and difficulties in understanding the questionnaire, while some suggested minor changes in words to enhance the clarity of the questionnaires. All patients’ suggestions and comments were taken into consideration and addressed for finalising the tool.

*Content validity*. The PSPSQ 2.0, in its original language, was already evaluated for content validity [1]. The questionnaire has already used by various experts to assess the satisfaction of the people on the pharmacists-delivered services [2, 18]. Hence, we assumed the tool was already validated for its content, and the questionnaire already contains items from the desired content domains. Therefore, we did not perform content validation on our own.

### Data collection

Eligible patients were approached for data collection. Respondents were asked to complete sociodemographic information and 20-item Arabic PSPSQ 2.0. Patients were given 20 minutes to complete the questionnaire by themselves. The study researcher assisted patients, if needed, while completing the questionnaire. Additional time was given to participants to complete the questionnaire if required. Additionally, patients were assured about the confidentiality of their information. The completed questionnaires were checked by the author and scoring was performed.

### Ethical consideration

This study was approved by institutional review boards from Princess Nourah bent Abdulrahman University (Approval # 20–0240), King Fahad Medical City (Approval # 20-388E) and University of Birmingham (Approval # ERN_20–0768).

Formal permission to translate, culturally adapt and validate the instrument PSPS 2.0 was granted before testing from authors of Sakharkar et al. (2015) [1]. Drs. Anandi V Law and Mark Bounthavong have the exclusive copyright for PSPSQ 2.0 and a non-commercial license agreement was signed.

Verbal and written consent was sought for all participants. All participants provided written informed consent in addition to initial verbal consent after providing them with a full explanation of the nature of the study procedure and before collecting data from them. Participants were assured that their participation in this study was voluntary, and confidentiality would be maintained.

### Statistical analysis

Data were cleaned, entered, and analysed using the SPSS 27. Socio-demographic data were analysed using descriptive statistics and the results are presented in terms of frequencies, percentages, and mean ± standard deviation (SD) or median and interquartile range [IQR] [31].

Psychometric analyses included testing for construct validity and reliability. The construct validity of the translated PSPSQ 2.0 was assessed using exploratory factor analysis (EFA) to extract the questionnaire component using the principal component (PC) for the items of each scale. Eigenvalues associated with each factor were analysed before extraction. Factors having more than 1 eigenvalue were extracted. A scree plot was further used to confirm the number of factors. Cronbach’s alpha coefficient was also used to measure the internal consistency and reliability of the dimensions of the questionnaire. It ranges from 0 to 1 for a completely unreliable test to for a completely reliable test respectively [26]. For the reliability test in this questionnaire, Cronbach’s alpha coefficient ≥ 0.70 was applied [32, 33].

## Results

### Participants characteristics

Table 2 depicts the characteristics and descriptive information of the 129 respondents. Out of 129 patients, 88 (68.22%) were male. The mean (±SD) age was 50 (±11.94) years. Almost a quarter 31 (24.03%) of the participants had an educational level of Diploma/high school, which was followed by a Bachelor’s degree level or higher 29 (22.48%). More than half of the participants 73 (56.59%) had insurance coverage by company and one third of participants 42 (32.56%) had monthly income less than 5000 Saudi Riyal.

**Table 2:** Sociodemographic characteristics of respondents (n = 129).

Study characteristics	Frequency (N = 129)	Percent (%)
**Gender,**		
** Male**	88	68.2
** Female**	41	31.8
**Age (year), mean ± (SD)**	50.5 ± (11.9)	
**Nationality,**		
** Saudi**	44	34.1
** Non- Saudi**	85	65.9
**Education,**		
** Illiterate**	14	10.9
** Elementary**	27	20.9
** Intermediate**	28	21.7
** Diploma/high school**	31	24
** Bachelor’s degree or higher**	29	22.5
**Income range (SR),**		
** <5000**	42	32.6
** 5000- <10000**	34	26.4
** 10000- <15000**	30	23.3
** ≥ 15000**	23	17.8
**Insurance coverage,**		
** Governmental**	14	10.9
** Insurance company**	73	56.6
** None**	42	32.6
**BMI (kg/m2), median [IQR]**	29.55 [26.2, 33.3]	
**Family history,**		
** None**	27	20.9
** DM**	34	26.4
** HTN**	4	3.1
** DM & HTN**	21	16.3
** DM & DLD**	7	5.4
** HTN & DLD**	1	0.8
** DM, HTN &DLD**	35	27.1
**MARS-5,**		
** Not adhere**	95	73.6
** Adhere**	34	26.4
**DDS,**		
** < 2.0 (little or no distress)**	32	24.8
** 2.0–2.9 (moderate distress)**	52	40.3
** > 3.0 (high distress)**	45	34.9
**HbA1c (%), median [IQR]**	9.8 [8.9, 11.2]	
**RBG mg/dl, median [IQR]**	190 [135, 276]	
**FBG mg/dl, median [IQR]**	238.95 [169.1, 293]	
**Diabetes duration year, median [IQR]**	8 [4, 14]	
**Number of comorbidities,**		
** None**	56	43.4
** 1**	42	32.6
** 2**	19	14.7
** 3**	7	5.4
** 4**	4	3.1
** 5**	0	0
** 6**	0	0
** 7**	1	0.8
**Number of medications, median [IQR]**	5 [4, 7]	

The median duration of diabetes was 8 [IQR 4, 14] years with a median HbA1c of 9.8% [IQR 8.9, 11.2]. Patients had up to seven comorbid conditions, with one chronic illness being the most observed 42 (32.56%) per participant, the mean number of comorbidities was 1 (SD ±1.2). Patients had a median of 5 medications [IQR 4, 7].

### Translation and cultural adaptation

The process of translation and cultural adaptation generated the Arabic version of PSPSQ 2.0 **(S2 File).** During the process, no significant difficulties were found. However, only a minor formatting problem was noted and relevant changes to a few items were made with some negligible changes in grammatical structures. The pilot testing showed that all participants could easily understand the preliminary version of the questionnaire. The study team proceeded with cultural adaptation.

### Validation and reliability analysis

For reliability testing and factor analysis, all survey items were included and analysed for the items in each of the three domains of PSPSQ 2.0 separately. The results of reliability and factor analysis are presented in detail below.

#### Domain (1): Quality of care

The sample adequacy using KMO was 0.924 which is considered ‘very good’, and Bartlett’s Test of Sphericity (3092.129) was significant (p-value<0.001), Table 3. All items of quality of care were allocated into one factor which explained 92.74% of variation in the data. The values of factor loading indicated that all times strongly belonged to the dimension of Quality of care. Cronbach’s alpha was 0.99, demonstrating high internal consistency (**S3 File).**

**Table 3:** Results of principal component analysis for quality of care.

Item	Factor loading
Quality of Care 1	0.982
Quality of Care 6	0.982
Quality of Care 10	0.981
Quality of Care 4	0.981
Quality of Care 8	0.976
Quality of Care 2	0.971
Quality of Care9	0.965
Quality of Care 7	0.964
Quality of Care3	0.952
Quality of Care 5	0.872

#### Domain (2): Interpersonal relationship

The sample adequacy using KMO was 0.848 which is considered ‘very good’, and Bartlett’s Test of Sphericity (905.74) was significant (p <0.001), Table 4. All items of interpersonal relationship were allocated into one factor which explained 80.54% of variation in the data. The values of factor loading indicated that all times strongly belonged to the dimension of interpersonal relationship. Cronbach’s alpha was 0.95, demonstrating high internal consistency (**S4 File)**.

**Table 4:** Results of principal component analysis for interpersonal relationship.

Item	Factor loading
Interpersonal Relationship 13	0.931
Interpersonal Relationship 15	0.924
Interpersonal Relationship 16	0.912
Interpersonal Relationship 12	0.908
Interpersonal Relationship 11	0.889
Interpersonal Relationship 14	0.816

#### Domain (3): Overall care

The sample adequacy using KMO was 0.777 which is considered ‘very good’, and Bartlett’s Test of Sphericity (602.582) was significant (p-value<0.001), Table 5. All items of overall care were allocated into one factor which explained 96.21% of variation in the data. The values of factor loading indicated that all times strongly belonged to the dimension of overall care. Cronbach’s alpha was 0.98, demonstrating high internal consistency (**S5 File)**.

**Table 5:** Results of principal component analysis for overall care.

Item	Factor loading
Overall18	0.986
Overall 19	0.982
Overall17	0.975

## Discussion

In general, assessing patient satisfaction with community pharmacy services is regarded as a crucial indicator of pharmacy service quality, particularly when introducing new pharmacist-delivered services [34]. The availability of a valid and reliable instrument will assist health professionals and pharmacists in identifying potential areas for service improvement and health expenditure, as well as in optimizing patient-guided planning and evaluation. Unfortunately, there was no such instrument to assess Arabic-speaking patients’ satisfaction with pharmacists’ services. In this study, the widely used Patient Satisfaction with Pharmacy Services (PSPS 2.0) tool, validated in various languages and settings [1, 2, 18], was translated, culturally adapted, and validated for the Arabic context to assess the satisfaction of Arabic-speaking chronic disease patients with community pharmacy services.

The three domains of PSPSQ 2.0 were reviewed and analysed separately: quality of care, interprofessional relationship, and overall care. The sample adequacy was 0.924, 0.848, and 0.777, respectively. Bartlett’s Test of Sphericity was significant overall dimensions. Exploratory factor analysis revealed validity of 92.7%, 80.5%, and 96.2%, respectively. The values of Cronbach’s alpha were 0.94, 0.92, and 0.84, respectively. High factor loadings indicate that questionnaire items are strongly correlated with their underlying constructs, suggesting good construct validity and relevance, and ensuring the items effectively capture the core aspects of the constructs. Furthermore, high Cronbach’s alpha coefficients signify that the questionnaire items within each factor are consistently measuring the same underlying construct, thus affirming the instrument’s reliability.

There were notable variations between the original English and the Arabic versions of the tool. Cronbach’s alpha was found to be greater than what the original instrument’s developer reported. In addition, the original study was conducted at the Department of Veterans Affairs and community-based clinics, whereas our study was conducted at an MTM clinic in a community pharmacy context [1]. Compared to studies that translated the PSPSQ 2.0 [2, 18], Cronbach’s alpha value in the three domains were higher in our study. Furthermore, Hassali et al. had four of the survey items excluded due to their low-reliability values (<0.50) [2]. The differences in Cronbach’s alpha values between our study and previous research can be attributed to several factors stemming from variations in study settings, population characteristics, and cultural contexts.

The study has some limitations that should be mentioned. First, this study was limited by patients’ typical perceptions of pharmacist service, in which they carried forward their past medication and counselling experiences to the present. This is especially significant because the MTM service provided these patients with novel clinical pharmacy services. Second, this study included patients with specific sociodemographic characteristics who visited one community pharmacy, in a specific region of Riyadh and may not be a true representation of patients from all over the country who live in different administrative sectors with varying geography and cultural backgrounds visiting community pharmacies. Thus, including patients from Saudi Arabia’s diverse cultural communities and environments in future studies provides more opportunities to test the instrument. Third, the study only included patients who received services from a single community pharmacy and did not include patients who visited other levels of healthcare institutions, such as a hospital pharmacy. Lastly, there is a risk of reporting bias since the questionnaire used in the study is a self-reporting tool.

It has been noted that the number of articles published, in Arabic-speaking countries, related to patient satisfaction has increased in recent years, although there are methodological deficiencies in the development of instruments to measure patient satisfaction. This research fulfils the need for the comprehensive, reliable and valid instrument for assessing patient satisfaction with pharmacy services using the Arabic version to be used in a regular way, not only to obtain results for research, but also to evaluate the impact of everyday practice on health care quality. In the future, this tool can be further validated to assess patients’ satisfaction with services provided by pharmacists in the hospital pharmacy setting.

## Conclusions

The Arabic PSPSQ 2.0 is a reliable and valid instrument to assess patient satisfaction with clinical services provided by community pharmacists. Given the critical importance of quality evidence in ensuring sustainability of rapidly expanding pharmacy services in the Arab world, the culturally adapted pharmacy satisfaction questionnaire will become an increasingly vital tool for evaluating and enhancing patient experiences and optimise pharmacy services. Further validation studies are recommended to be undertaken in other pharmacy settings and among diverse Arabic communities to strengthen its validity and relevance.

## Supporting information

S1 File PSPSQ 2.0 (English version). (PDF)

S2 File PSPSQ 2.0 (Arabic version). (PDF)

S3 File Quality of care. (PDF)

S4 File Interpersonal relationship. (PDF)

S5 File Overall care. (PDF)
