# Supplementary material for: Translation, cultural adaptation and validation of Patient Satisfaction with Pharmacist Services Questionnaire (PSPSQ) 2.0 into the Arabic language among people with diabetes
Source: PLoS One. 2024 Jun 27;19(6):e0298848. doi: 10.1371/journal.pone.0298848 (PMC11210780; doi:10.1371/journal.pone.0298848)
Supplement: S1 File — (PDF) [file pone.0298848.s001.pdf]

S1 File. PSPS 2.0 Questionnaire (English Version)

Please complete this survey by checking the option that best describes your opinion:

	**Quality of Care**	Strongly Agree	Agree	Disagree	Strongly Disagree
1	The pharmacist fully addressed the main health reason/concerns/issues during my visit.	4	3	2	1
2	The pharmacist was professional in all of our interactions.	4	3	2	1
3	The pharmacist explained information to me in a manner that I could understand.	4	3	2	1
4	The pharmacist checked to see if I understood all the information.	4	3	2	1
5	The pharmacist spent as much time necessary to help me with my questions and concerns.	4	3	2	1
6	The pharmacist made sure I understood how important it is to follow the drug regimen.	4	3	2	1
7	The pharmacist provided useful recommendations on how to take my medications.	4	3	2	1
8	The pharmacist provided useful recommendations about managing my overall health (e.g. diet, exercise).	4	3	2	1
9	The pharmacist worked with me to manage my medication related issues (e.g. cost, side effects of drugs).	4	3	2	1
10	The pharmacist followed up on my progress in a timely manner.	4	3	2	1

	**Interpersonal Relationship (pharmacist/patient)**	Strongly Agree	Agree	Disagree	Strongly Disagree
11	The pharmacist was caring and kind in dealing with my health issues.	4	3	2	1
12	The pharmacist encouraged me to achieve my treatment goals.	4	3	2	1
13	I felt comfortable in my interactions with the pharmacist.	4	3	2	1
14	The pharmacist was respectful to me during our interactions.	4	3	2	1
15	The pharmacist was committed to improving my health.	4	3	2	1
16	I could trust the information that the pharmacist provided.	4	3	2	1

	**Overall**	Strongly Agree	Agree	Disagree	Strongly Disagree
17	I was satisfied with the overall care provided by my pharmacist.	4	3	2	1
18	I would recommend my pharmacist to people I know.	4	3	2	1
19	If needed, I would continue seeing this pharmacist for my healthcare needs.	4	3	2	1

20	The overall care provided by the pharmacist.	Exceeded my expectations 4	Met my expectations 3	Did not meet my expectations 2	Had no expectations 1

Copyrighted Law and Bounthavong 2007 revised 2014
