# Supplementary material for: Translation, cultural adaptation and validation of Patient Satisfaction with Pharmacist Services Questionnaire (PSPSQ) 2.0 into the Arabic language among people with diabetes
Source: PLoS One. 2024 Jun 27;19(6):e0298848. doi: 10.1371/journal.pone.0298848 (PMC11210780; doi:10.1371/journal.pone.0298848)
Supplement: S2 File — (PDF) [file pone.0298848.s002.pdf]

**S2 File. PSPS 2.0 Questionnaire (Arabic Version)**

استبيان رضا المريض عن الخدمات الصيدلية

يرجى إكمال هذا الاستبيان عن طريق تحديد الخيار المناسب لرأيك.

| | جودة الرعاية الصحية | أوافق بشدة | أوافق | لا اوافق | لا أوافق بشدة |
|---|---|---|---|---|---|
| 1 | تطرق الصيدلي بشكل كامل للأسباب / المخاوف / المشاكل الصحية خلال الزيارة | 4 | 3 | 2 | 1 |
| 2 | كان الصيدلي على قدر عالي من المهنية في التعامل معي | 4 | 3 | 2 | 1 |
| 3 | شرح الصيدلي جميع المعلومات بأسلوب أستطيع فهمه | 4 | 3 | 2 | 1 |
| 4 | تأكد الصيدلي من فهمي للمعلومات | 4 | 3 | 2 | 1 |
| 5 | قضى الصيدلي الوقت الكافي لمساعدتي في أسئلتي ومخاوفي | 4 | 3 | 2 | 1 |
| 6 | تأكد الصيدلي من فهمي لطريقة أخذ الدواء | 4 | 3 | 2 | 1 |
| 7 | قدم الصيدلي توصيات مفيدة لكيفية أخذ أدويتي. | 4 | 3 | 2 | 1 |
| 8 | قدم الصيدلي توصيات مفيدة عن صحتي (مثل النظام الغذائي والتمارين الرياضية) | 4 | 3 | 2 | 1 |
| 9 | عمل الصيدلي معي لحل المشاكل المتعلقة بأدويتي (مثل التكلفة والآثار الجانبية للأدوية) | 4 | 3 | 2 | 1 |
| 10 | تابع الصيدلي تطور حالتي الصحية في أوقات مناسبة | 4 | 3 | 2 | 1 |

| | العلاقة الشخصية (بين الصيدلي / المريض) | أوافق بشدة | أوافق | لا اوافق | لا أوافق بشدة |
|---|---|---|---|---|---|
| 11 | كان الصيدلي مهتم ولطيف في التعامل مع مشاكلي الصحية | 4 | 3 | 2 | 1 |
| 12 | شجعني الصيدلي على تحقيق أهدافي العلاجية | 4 | 3 | 2 | 1 |
| 13 | شعرت بالراحة أثناء تعاملي مع الصيدلي | 4 | 3 | 2 | 1 |
| 14 | كان الصيدلي محترما أثناء التعامل معي | 4 | 3 | 2 | 1 |
| 15 | كان الصيدلي ملتزمًا بتحسين صحتي | 4 | 3 | 2 | 1 |
| 16 | أثق بالمعلومات التي قدمها الصيدلي. | 4 | 3 | 2 | 1 |

| | الكل | أوافق بشدة | أوافق | لا اوافق | لا أوافق بشدة |
|---|---|---|---|---|---|
| 17 | كنت راضيًا عن الرعاية الشاملة التي قدمها الصيدلي | 4 | 3 | 2 | 1 |
| 18 | سأنصح غيري بالتعامل مع هذا الصيدلي | 4 | 3 | 2 | 1 |
| 19 | إن دعت الحاجة سأستمر في المتابعة مع هذا الصيدلي في ما يخص احتياجاتي الصحية | 4 | 3 | 2 | 1 |

| | | | | | |
|---|---|---|---|---|---|
| 20 | الرعاية الشاملة التي قدمها الصيدلي | فاقت توقعاتي<br>4 | مثل توقعاتي<br>3 | لم تلب توقعاتي<br>2 | لم يكن لدي توقعات<br>1 |

Copyrighted Law and Bounthavong 2007 revised 2014
