# Supplementary material for: Translation, cultural adaptation and validation of Patient Satisfaction with Pharmacist Services Questionnaire (PSPSQ) 2.0 into the Arabic language among people with diabetes
Source: PLoS One. 2024 Jun 27;19(6):e0298848. doi: 10.1371/journal.pone.0298848 (PMC11210780; doi:10.1371/journal.pone.0298848)

S3 File. Quality of care

Total Variance Explained

Component	Initial Eigenvalues			Extraction Sums of Squared Loadings		
	Total	% of Variance	Cumulative %	Total	% of Variance	Cumulative %
1	9.274	92.740	92.740	9.274	92.740	92.740
2	.330	3.304	96.045			
3	.104	1.038	97.083			
4	.100	.998	98.081			
5	.054	.542	98.623			
6	.041	.411	99.034			
7	.037	.367	99.401			
8	.028	.278	99.679			
9	.024	.238	99.916			
10	.008	.084	100.000			

Extraction Method: Principal Component Analysis.

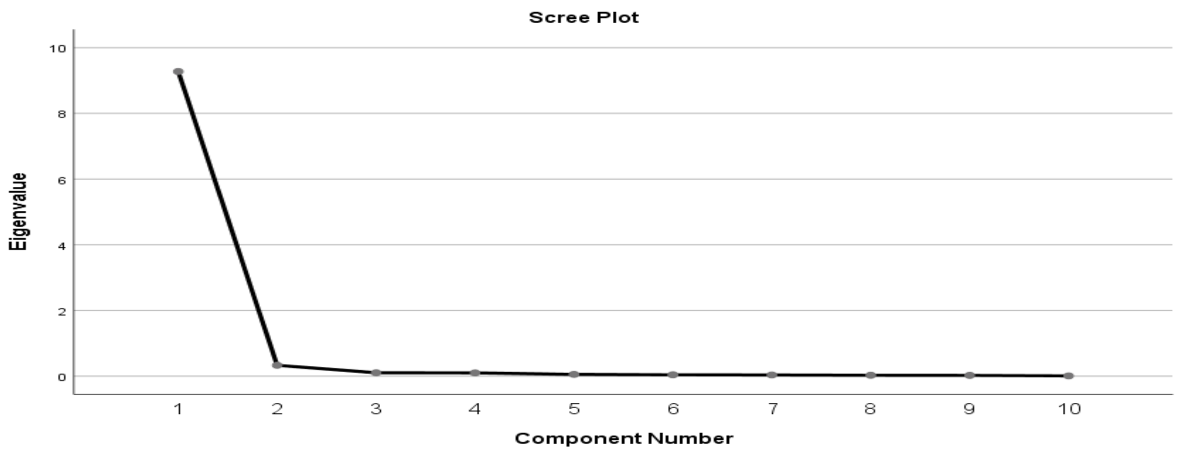

Supplement: S3 File — (PDF) [file pone.0298848.s003.pdf]
