# Supplementary material for: Translation, cultural adaptation and validation of Patient Satisfaction with Pharmacist Services Questionnaire (PSPSQ) 2.0 into the Arabic language among people with diabetes
Source: PLoS One. 2024 Jun 27;19(6):e0298848. doi: 10.1371/journal.pone.0298848 (PMC11210780; doi:10.1371/journal.pone.0298848)

S4 File. Interpersonal relationship

Total Variance Explained

Component	Initial Eigenvalues			Extraction Sums of Squared Loadings		
	Total	% of Variance	Cumulative %	Total	% of Variance	Cumulative %
1	4.832	80.541	80.541	4.832	80.541	80.541
2	.617	10.289	90.830			
3	.215	3.582	94.412			
4	.167	2.775	97.187			
5	.104	1.731	98.918			
6	.065	1.082	100.000			

Extraction Method: Principal Component Analysis.

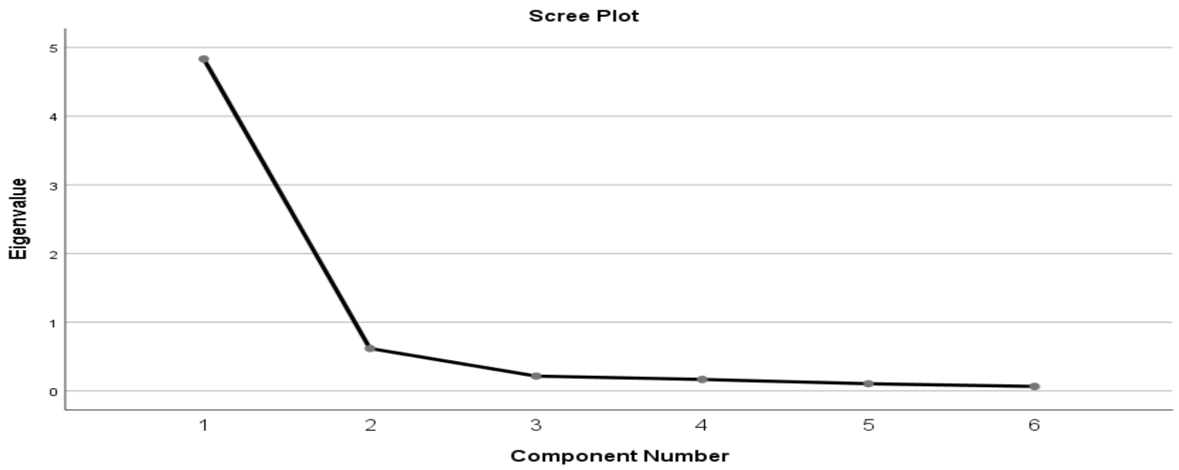

Supplement: S4 File — (PDF) [file pone.0298848.s004.pdf]
