# Supplementary material for: Translation, cultural adaptation and validation of Patient Satisfaction with Pharmacist Services Questionnaire (PSPSQ) 2.0 into the Arabic language among people with diabetes
Source: PLoS One. 2024 Jun 27;19(6):e0298848. doi: 10.1371/journal.pone.0298848 (PMC11210780; doi:10.1371/journal.pone.0298848)

**S5 File. Overall care**

**Total Variance Explained**

| Component | Initial Eigenvalues | | | Extraction Sums of Squared Loadings | | |
|---|---|---|---|---|---|---|
| | Total | % of Variance | Cumulative % | Total | % of Variance | Cumulative % |
| 1 | 2.886 | 96.205 | 96.205 | 2.886 | 96.205 | 96.205 |
| 2 | .075 | 2.494 | 98.699 | | | |
| 3 | .039 | 1.301 | 100.000 | | | |

Extraction Method: Principal Component Analysis.

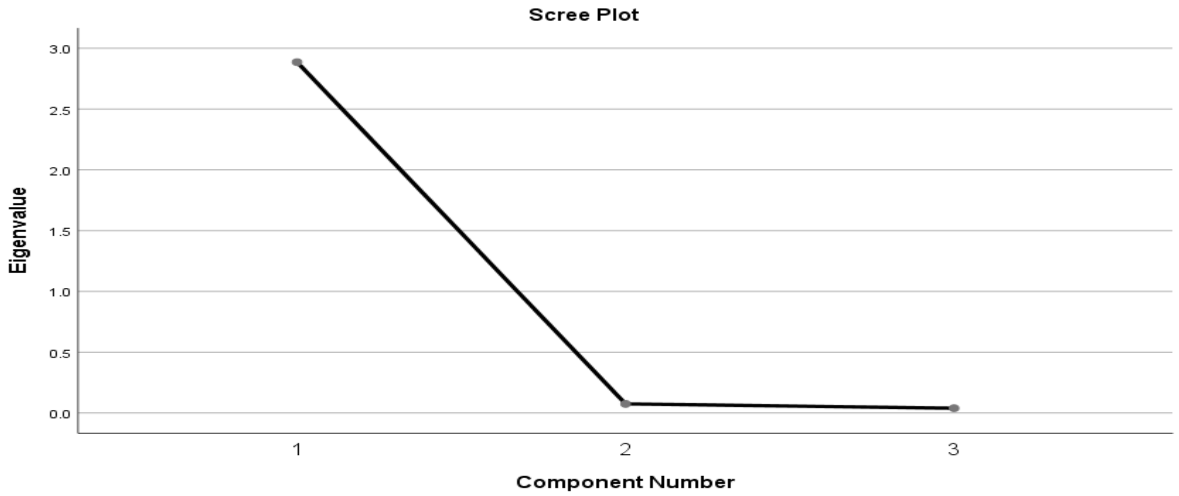

Supplement: S5 File — (PDF) [file pone.0298848.s005.pdf]
